# Corticosteroids in immunocompromised ICU patients with pneumonia: risks, evidence, and clinical practice

**DOI:** 10.1186/s41479-026-00202-5

**Published:** 2026-06-25

**Authors:** Jeison Andrés Morales-Olivera, Paula O. Narvaez-Ramirez, Ingrid G. Bustos, Lina F. Martinez-Lemus, Natalia Sanabria-Herrera, Cristian C. Serrano-Mayorga, Valeria Aguirre Gutierrez, Alex Julián Forero-Delgadillo, Lina Mar Mendez-Castillo, Gina Sofia Montaño, Luis Felipe Reyes

**Affiliations:** 1 Clínica Universidad de La Sabana, Chía, Colombia; 2 Unisabana Center for Translational Science, School of Medicine, Universidad de La Sabana, Chía, Colombia; 3 Biosciences PhD program, School of Engineering, Universidad de La Sabana, Chía, Colombia; 4 Doctorado en Ciencias Clínicas, Facultad de Medicina, Universidad de La Sabana, Chía, Colombia; 5 Pandemic Sciences Institute, University of Oxford, Oxford, UK; 6 Clinical and Translational Medicine Research Group, Critical Physiology Subdivision, Intensive Care Department, Hospital Federico Lleras Acosta, Ibagué, Colombia; 7 Intensive Care Department, Hospital Federico Lleras Acosta, Ibagué, Colombia; 8 School of Medicine, Corporación Institución Universitaria Salud Colombia, Ibagué, Colombia; 9 Universidad de La Sabana, Campus Puente del Común, KM 7.5 Autopista Norte de Bogotá, Chía, Colombia

**Keywords:** Immunocompromised host, Pneumonia, Intensive care units, Glucocorticoids, Opportunistic infections, Community-acquired pneumonia

## Abstract

Adjunctive corticosteroids are increasingly used in Severe Community-Acquired Pneumonia (sCAP) requiring admission to the intensive care unit (ICU), based on evidence derived largely from immunocompetent populations. Immunocompromised hosts, however, represent a distinct and growing subgroup among critically ill patients with sCAP, characterised by baseline immune defects, broader pathogen spectra, and a heightened susceptibility to treatment-related harm. Whether corticosteroid strategies validated in the general population can be safely and effectively extrapolated to immunocompromised ICU patients with sCAP remains uncertain, despite their growing use in contemporary ICU practice. Immunocompromised patients admitted to the ICU with sCAP experience compounded alterations in host defence, driven by pre-existing immune dysfunction and superimposed critical illness–associated immune dysregulation. In this setting, corticosteroids may attenuate inflammation-mediated lung injury, but may also deepen immune suppression, distort clinical evolution, and increase susceptibility to opportunistic superinfections and pathogen reactivation. Randomised trials and meta-analyses supporting corticosteroid use in sCAP have systematically excluded immunocompromised patients, treating immunosuppression as an exclusion criterion rather than a stratification variable, and commonly rely on short-term endpoints that fail to capture delayed infectious complications relevant to this population. Available observational data and pathogen-specific evidence suggest substantial heterogeneity of treatment effect (HTE) across immunocompromised subgroups, influenced by immune substrate, pathogen context, and cumulative immunosuppressive burden. Among the pathogen-specific contexts in which corticosteroids have the strongest evidence, *Pneumocystis jirovecii* pneumonia (PJP) stands out as the principal indication: adjunctive corticosteroids have demonstrated clear mortality benefit in AIDS-associated severe PJP, though this benefit does not extend uniformly to HIV-negative immunocompromised patients with PJP. Consequently, standard corticosteroid treatment in all CAP subgroups is unlikely to be appropriate. In immunocompromised ICU patients with sCAP, corticosteroid therapy should neither be routinely applied nor categorically avoided. Instead, decisions should be individualised, guided by a clearly defined therapeutic target, careful assessment of immune status, and consideration of the risk for opportunistic infections. When used, corticosteroids should be administered at the lowest effective dose and for the shortest feasible duration. Dedicated studies incorporating immune stratification, pathogen-informed approaches, and outcomes relevant to delayed infections are urgently needed. Until such evidence is available, careful risk-balance assessments to decide steroids in these patients.

## Background

Lower respiratory infections (LRIs), including pneumonia, continue to impose a substantial global burden despite advances in vaccination, antimicrobial therapy, and critical care [1]. According to the Global Burden of Disease (GBD) 2023, the estimated global incidence of LRIs in 2021 was 242 million incident episodes, corresponding to 3,006 episodes per 100,000 population. In the same year, non–COVID-19 LRIs were responsible for 2.50 million deaths, equivalent to 31 deaths per 100,000 population, remaining the leading cause of infection-related mortality worldwide [2]. Beyond its population-level impact, community-acquired pneumonia (CAP) remains a major driver of acute-care utilization. Contemporary data indicate that approximately 20–25% of patients hospitalised with CAP develop severe disease requiring ICU admission, where the need for invasive mechanical ventilation (IMV) or vasopressor support is common and short-term mortality remains frequent [3]. These observations underscore that sCAP continues to represent a high-risk syndrome with significant residual mortality.

Immunocompromised patients represent a substantial and clinically distinct subgroup of CAP. In a secondary analysis from the Global Initiative for Methicillin-resistant *Staphylococcus aureus* Pneumonia (GLIMP) study, including adults hospitalised with CAP, 18% of 3,702 patients had at least one immunocompromising condition, most commonly chronic corticosteroid use, haematological malignancy, or active cancer therapy [4]. ICU-specific data reports even higher proportions, with up to 39% of CAP patients meeting immunocompromise criteria at admission [5]. This population consistently experiences higher in-hospital mortality (24.4% vs. 14.4%) and persistently increased mortality at 6 months (27.4% vs. 17.4%) and 12 months (8.1% vs. 6.4%) following discharge, compared with non-immunocompromised patients [6]. Greater etiologic diversity, with more frequent identification of opportunistic pathogens, further underscores the diagnostic and therapeutic challenges of sCAP in immunocompromised hosts [4, 7–10].

In parallel, systemic corticosteroids have re-emerged as a central therapy in sCAP following recent randomized trials and meta-analyses conducted largely in non-immunocompromised populations [11–13]. In the CAPE COD trial, early intravenous hydrocortisone in ICU patients with sCAP significantly reduced 28-day mortality (6.2% vs. 11.9%) and decreased the need for endotracheal intubation (18.0% vs. 29.5%) and vasopressor initiation (15.3% vs. 25.0%) among patients not receiving these therapies at baseline [3]. A recent systematic review and dose–response meta-analysis of 18 randomised trials (4,661 patients) similarly found that corticosteroids probably reduce mortality in sCAP and lower the risk of invasive mechanical ventilation, at the cost of increased hyperglycemia [14]. However, the generalizability of these findings to immunocompromised ICU patients remains uncertain, as this population has been largely excluded from pivotal trials, and short-term endpoints may not capture delayed infectious complications or other harms relevant to impaired host defences.

For the purposes of this review, immunocompromised patients are defined as adults with pre-existing conditions or therapies that impair innate and/or adaptive immune responses at the time of ICU admission. These include hematologic or solid malignancies and their treatments, solid organ or hematopoietic stem cell transplantation, advanced HIV infection, primary immunodeficiencies, and exposure to systemic corticosteroids or other immunosuppressive or immunomodulatory agents, in line with contemporary American Thoracic Society consensus efforts to standardise definitions in immunocompromised host pneumonia [7]. Table 1 summarises the major categories of immunocompromise relevant to ICU pneumonia, including baseline immune defects, common opportunistic pathogens, and corticosteroid-associated risks specific to each category.

**Table 1:** Characterization of immunocompromised hosts: baseline immune defects, pathogen susceptibility, and corticosteroid-associated risks

Type of Immunocompromise	Primary Immune Defect	Common Opportunistic Pathogens	Corticosteroid-Associated Risks
Hematologic malignancy and neutropenia	Neutropenia (< 500/µL), lymphopenia, impaired cell-mediated immunity, mucosal barrier disruption	Gram-negative bacteria (Pseudomonas, Enterobacteriaceae), Aspergillus spp., Candida spp., Mucorales	Invasive fungal infection (IPA, candidemia), bacterial breakthrough, diagnostic masking
Solid organ transplant	T-cell dysfunction (calcineurin inhibitors), combined immunosuppression, impaired antigen presentation	CMV, Aspergillus spp., Nocardia, Pneumocystis jirovecii, Toxoplasma gondii	Viral reactivation (CMV, HSV, HBV), invasive fungal infection, cumulative immunosuppression
Hematopoietic stem cell transplant (allogeneic)	Profound lymphopenia, GVHD-related immune dysfunction, mucosal barrier disruption, delayed immune reconstitution	Aspergillus spp., CMV, HSV, VZV, adenovirus, Gram-negative bacteria, Pneumocystis jirovecii	Severe invasive fungal infection, CMV disease, viral reactivation syndromes, GVHD exacerbation
HIV/AIDS (CD4 < 200 cells/µL)	CD4 + T-cell depletion, impaired Th1 response, reduced cell-mediated immunity	Pneumocystis jirovecii, Cryptococcus neoformans, Mycobacterium tuberculosis, atypical mycobacteria, CMV, Toxoplasma gondii	Opportunistic infection progression, immune reconstitution inflammatory syndrome (IRIS) if antiretroviral therapy initiated
Chronic corticosteroid therapy	Impaired phagocytosis, lymphopenia, HPA axis suppression, reduced cytokine production (IL-2, IFN-γ)	Pneumocystis jirovecii, Strongyloides stercoralis, Aspergillus spp., Mycobacterium tuberculosis, atypical bacteria	Cumulative dose effect, Strongyloides hyperinfection syndrome, Pneumocystis pneumonia, invasive aspergillosis
Biologic and targeted immunomodulatory therapies	Variable: TNF-α blockade (anti-TNF), IL-6 inhibition, JAK-STAT pathway inhibition, CD20 depletion (rituximab)	Anti-TNF: Mycobacterium tuberculosis, Histoplasma capsulatum; Anti-CD20: Pneumocystis jirovecii, HBV reactivation; JAK inhibitors: Herpes zoster, atypical infections	Agent-specific risks, reactivation syndromes, synergistic immunosuppression when combined with corticosteroids
Solid malignancy (advanced/metastatic)	Lymphopenia, impaired cell-mediated immunity, malnutrition-associated immune dysfunction, chemotherapy-induced neutropenia	Bacterial pneumonia, Pneumocystis jirovecii (with chemotherapy), Aspergillus spp., atypical pathogens	Invasive fungal infection, bacterial superinfection, diagnostic delay, poor functional reserve

Abbreviations: CMV, cytomegalovirus; GVHD, graft-versus-host disease; HBV, hepatitis B virus; HPA, hypothalamic-pituitary-adrenal; HSV, herpes simplex virus; IFN-γ, interferon-gamma; IL-2, interleukin-2; IPA, invasive pulmonary aspergillosis; JAK-STAT, Janus kinase-signal transducer and activator of transcription; TNF-α, tumour necrosis factor-alpha; VZV, varicella-zoster virus

This table synthesizes baseline immune defects and corticosteroid-associated risks across major categories of immunocompromise relevant to ICU pneumonia. Pathogen susceptibility patterns reflect both baseline host vulnerability and amplified risk under additional corticosteroid exposure. Risk profiles are not mutually exclusive and may overlap in patients with combined immunodeficiencies or multiple immunosuppressive exposures

In this context of limited strong evidence, frequent trial exclusions, and marked biological heterogeneity, the central clinical challenge is not whether corticosteroids are effective in sCAP in general, but whether specific subgroups of immunocompromised ICU patients with sCAP may derive net benefit or experience disproportionate harm. Accordingly, this review synthesises the best available evidence to critically examine corticosteroid therapy in immunocompromised ICU patients with sCAP, focusing on who might benefit, under what circumstances, and with which safeguards.

## Immunocompromised patients with pneumonia in the ICU

### Baseline immunosuppression and host vulnerability

Immunocompromised patients admitted to the ICU with CAP have heterogeneous, biologically relevant defects in host defence that are present at the time of infection. Contemporary consensus frameworks emphasise defining immunocompromise by functional immune impairment at clinical presentation rather than by diagnostic labels alone, as distinct immunosuppressive states converge on different failure points of antimicrobial immunity [7]. These baseline abnormalities determine not only pathogen susceptibility but also the immune reserve available to respond to severe infection and subsequent critical illness [15, 16].

At the level of innate immunity, baseline immunosuppression may involve quantitative depletion or functional impairment of neutrophils and monocyte/macrophage lineages, leading to reduced phagocytosis, impaired oxidative burst, and diminished intracellular killing of bacteria and fungi [17, 18]. Mechanistic syntheses describe defective pattern-recognition receptor signalling, impaired coordination between innate sensing and adaptive responses, and reduced production of key pro-inflammatory mediators such as *interleukin-1β* (IL-1*β*) and Tumour Necrosis Factor*-α* (TNF*-α*) [19], which collectively compromise early microbial containment [20, 21]. Impaired antigen processing and presentation by mononuclear phagocytes limit effective priming of pathogen-specific T lymphocytes, weakening the transition from innate to adaptive immunity [22–24].

Adaptive immune dysfunction constitutes a second critical axis of vulnerability. Many immunocompromised states are characterised by quantitative and qualitative abnormalities of CD4⁺ and CD8⁺ T lymphocytes, including lymphopenia, impaired proliferative capacity, and reduced effector cytokine production, particularly interferon-γ (IFN*-γ*), which is essential for intracellular pathogen control [25–28]. Experimental and clinical data further characterise phenotypes consistent with T-cell exhaustion, characterised by diminished cytotoxic function and reduced responsiveness to antigenic stimulation, features particularly relevant to viral and opportunistic fungal pneumonias [29, 30]. Importantly, these functional defects may persist despite apparent recovery of lymphocyte counts, underscoring that immune competence cannot be inferred solely from routine laboratory parameters [31–33].

Together, these baseline abnormalities establish a host milieu characterised by impaired pathogen sensing, suboptimal effector responses, and limited immune reserve, predisposing immunocompromised patients to higher microbial burden, delayed clearance, and opportunistic infections. ICU epidemiologic data are consistent with this framework, demonstrating higher mortality and increased detection of pathogens such as *P.jirovecii* and *Aspergillus* spp. among immunocompromised patients with CAP compared with immunocompetent hosts [6, 34–36].

### Immune dysregulation induced by severe pneumonia and critical illness

Progression from pneumonia to critical illness introduces a second, superimposed layer of immune perturbation driven by systemic inflammation, tissue injury, and organ dysfunction. Within the Sepsis-3 framework, life-threatening infection is defined by organ dysfunction arising from a dysregulated host response [37], explicitly recognising that injurious inflammation and impaired antimicrobial defence may coexist rather than unfold as a linear sequence [38]. This conceptualisation is supported by studies demonstrating that severe infection induces complex and sustained alterations across immune compartments [39, 40].

At the innate immune level, critical illness is associated with impaired antigen presentation, altered myeloid differentiation, and functional reprogramming of monocytes and macrophages. At the adaptive level, apoptosis-driven depletion of CD4⁺ and CD8⁺ T cells, reduced T-cell receptor signalling, and diminished production of IFN-*γ* and *interleukin-2* (IL-2) have been documented, changes that are linked to secondary infections and viral reactivation [29, 41]. Immune profiling across heterogeneous aetiologies of severe injury further demonstrates convergence toward a shared signature of delayed acquired immunodeficiency, characterised by coordinated alterations in markers of antigen presentation and lymphocyte function [42, 43].

In immunocompromised hosts with pneumonia, ICU-acquired immune dysregulation does not replace baseline defects but rather superimposes additional functional impairments on an already compromised immune system [44]. Pre-existing abnormalities in pathogen recognition and effector responses coexist with critical illness–induced lymphocyte depletion, impaired antigen presentation, and suppression of key cytokine pathways, resulting in compounded deficits in pathogen control [29, 41, 42]. At the same time, inflammatory injury may persist locally within the lung [38, 39].

Biological heterogeneity further complicates this landscape. In acute respiratory distress syndrome (ARDS), latent class analyses have identified reproducible subphenotypes, including a hyperinflammatory phenotype marked by elevated inflammatory mediators and worse outcomes, alongside less inflammatory phenotypes with distinct clinical trajectories [45]. Although ARDS subphenotype are not equivalent to immunocompromised states, they reinforce a broader principle: critically ill patients segregate into biologically distinct host-response states, with important implications for immunomodulatory interventions [46–49].

### Corticosteroids as an additional immunologic modulator in critical illness

Within this already dysregulated immune environment, systemic corticosteroids act as an additional immunologic modulator, exerting pleiotropic effects through genomic and non-genomic mechanisms [50, 51]. Detailed analyses of glucocorticoid biology demonstrate suppression of pro-inflammatory gene transcription, including IL-*1β*, IL-6, and TNF-*α*, alongside modulation of leukocyte trafficking, antigen presentation, and lymphocyte survival in a cell-type–specific and context-dependent manner [52, 53]. These properties underpin the potential of corticosteroids to attenuate inflammation-driven lung injury, but also raise concern for impaired antimicrobial defence in hosts with limited immune reserve.

Critical illness further alters corticosteroid biology by dysregulating hypothalamic–pituitary–adrenal axis signalling, altering cortisol metabolism, and inducing tissue-level glucocorticoid resistance. The SCCM/ESICM review on critical illness–related corticosteroid insufficiency describes how systemic inflammation reduces cortisol clearance and modifies glucocorticoid receptor signalling, complicating the prediction of corticosteroid effects in critically ill patients [54]. In immunocompromised ICU patients with pneumonia, exogenous corticosteroids act in the context of baseline immune defects, ICU-acquired immune dysregulation, and altered corticosteroid responsiveness, rendering net immunologic effects highly context-dependent.

### Corticosteroid impact on the lung microbiome

Although underexplored and recently characterised, different studies have highlighted the symbiotic relationship between the lung microbiome and the immune system in maintaining pulmonary homeostasis and regulating immune responses against pathogens [55, 56]. Lung microbiome dynamics are associated with an increasing number of lung diseases, including asthma, chronic obstructive pulmonary disease, and Lower respiratory tract infections [56–59]. For example, among ventilated patients colonised with *Pseudomonas aeruginosa*, reduced lung microbiome diversity has been associated with pneumonia development [60]. Furthermore, the bacterial burden of pneumonia has been shown to correlate inversely with phylogenetic diversity of the lung microbiome [61, 62].

It therefore becomes essential to explore and understand the impact of corticosteroid use in lung microbiome to inform better clinical and therapeutic approaches. Currently, very limited data describing the effects of corticosteroids on the lung microbiome is available. A cohort study including 200 ICU-admitted patients compared microbiome diversity between individuals treated with hydrocortisone and those who did not receive corticosteroids. Distinct microbial patterns emerged between survivors and non-survivors, with the latter exhibiting higher abundances of Alphaproteobacteria and Bacilli, suggesting a potential association between microbial composition and corticosteroid treatment response. These findings further indicate a possible link between microbial dysbiosis, inflammation, and poor prognosis [63]. Evidence from a systematic analyses including five cohorts further supports the notion that corticosteroid therapy may influence respiratory microbiome diversity and composition, although findings remain heterogeneous across diseases, treatment routes, and study designs [58].

### Conceptual framework: the immune double hit and corticosteroid modulation

Taken together, immunocompromised ICU patients with pneumonia experience an immune double hit that is cumulative and mechanistically layered. The first hit consists of baseline immunosuppression, characterised by distinct defects in innate and adaptive immunity that limit effective antimicrobial defence from the onset of infection. The second hit arises from pneumonia- and critical illness–induced immune dysregulation, which further impairs pathogen clearance by depleting lymphocytes, dysregulating antigen presentation, and sustaining suppression of key effector pathways, while failing to fully abrogate injurious inflammatory responses [38, 41, 42].

When systemic corticosteroids are introduced into this context, they constitute a third, exogenous axis of immunologic modulation, interacting with both baseline and ICU-acquired immune defects. Through their broad effects on cytokine transcription, leukocyte trafficking, and antigen presentation, corticosteroids may attenuate inflammation-driven tissue injury in selected scenarios, but may also deepen immune suppression and further compromise pathogen control in others [52]. These effects are further shaped by critical illness–related alterations in cortisol metabolism and tissue glucocorticoid responsiveness, which may modify the magnitude and direction of corticosteroid effects at the cellular level [54].

**Fig. 1:**
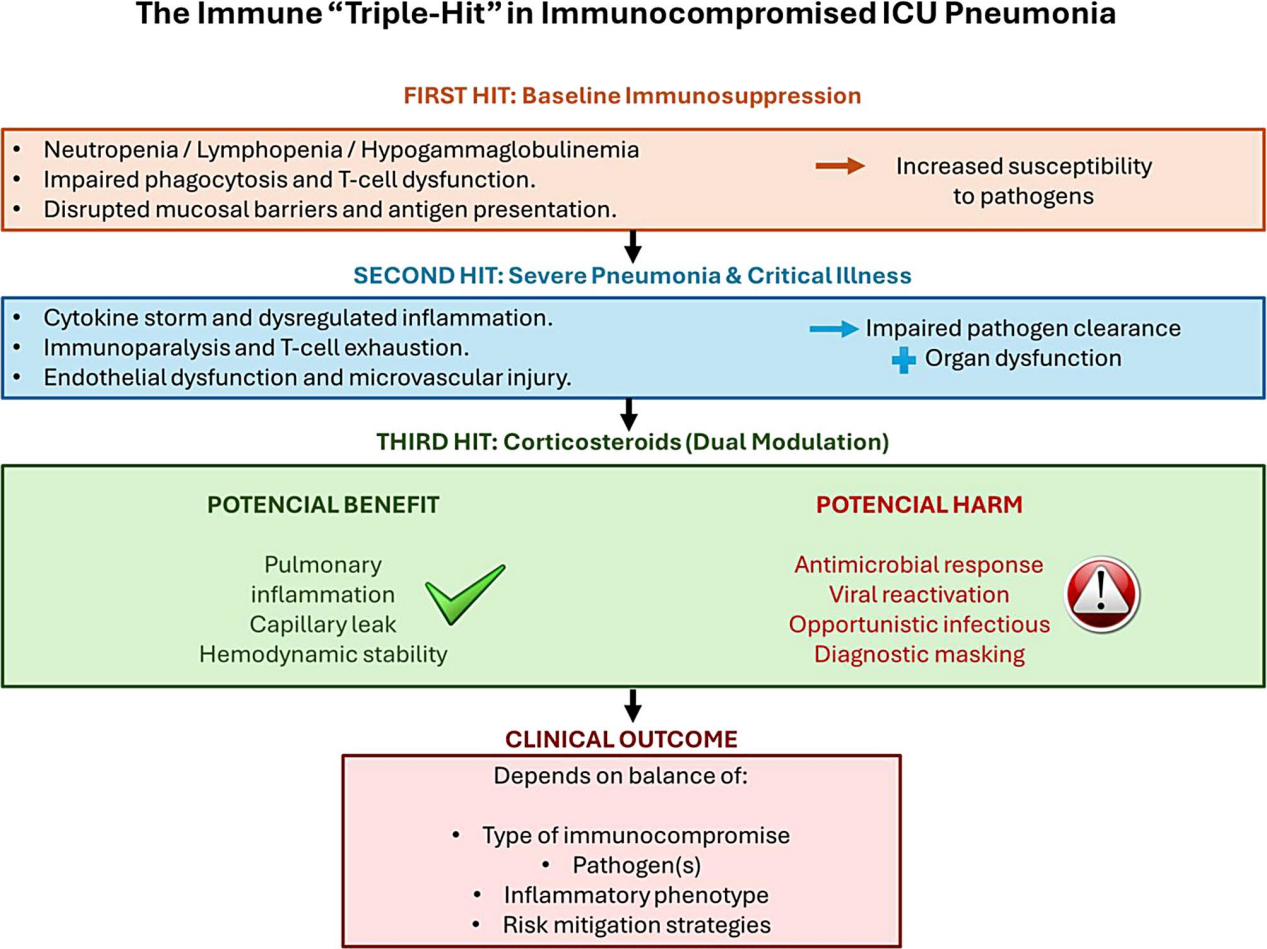
The Immune “Triple-Hit” Conceptual Model in Immunocompromised ICU Patients with Pneumonia. Immunocompromised patients admitted to the ICU with pneumonia experience compounded immune dysfunction through three sequential but overlapping processes. First, baseline immunosuppression (amber) impairs antimicrobial defence at the time of infection through neutropenia, lymphopenia, impaired phagocytosis, T-cell dysfunction, and disrupted mucosal barriers, collectively increasing susceptibility to pathogens and delaying microbial clearance. Second, severe pneumonia and critical illness (blue) impose additional immune dysregulation, characterised by a cytokine storm, immunoparalysis, T-cell exhaustion, and endothelial dysfunction, further impairing pathogen clearance and contributing to organ dysfunction. Third, corticosteroids (green) act as dual modulators: they may attenuate pulmonary inflammation, reduce capillary leak, and improve hemodynamic stability, but simultaneously deepen immune suppression, facilitate viral reactivation and opportunistic infections, and mask clinical signals that guide diagnostic decision-making. The clinical outcome (purple) depends on the complex interactions among the type and intensity of immunocompromise, pathogen context, host inflammatory phenotype, and the implementation of risk mitigation strategies, including screening, prophylaxis, and enhanced microbiologic surveillance. This framework clarifies why uniform corticosteroid strategies are unlikely to be appropriate in this biologically heterogeneous population, and why individualised decision-making anchored to explicit therapeutic targets and active risk mitigation is essential

This integrated framework clarifies why uniform corticosteroid strategies are biologically implausible in immunocompromised ICU patients with sCAP and underscores the need for individualised, pathophysiology-informed decision-making. Figure 1 illustrates this integrated conceptual framework, depicting the immune triple-hit experienced by immunocompromised ICU patients with sCAP and the dual nature of corticosteroid modulation within this already dysregulated immune environment.

## Rationale for corticosteroid use

### Mechanistic considerations

Corticosteroids are frequently considered in sCAP because they can, *in principle*, modulate host response pathways that contribute to lung injury, gas exchange impairment, and shock physiology in critical illness. At the molecular level, glucocorticoids exert their effects through the GR, a ligand-activated transcription factor that regulates gene expression by directly binding to glucocorticoid response elements, interacting with negative response elements, and *tethering* to other transcription factors central to inflammatory signalling, including NF-κB and AP-1 [52, 64]. Through these mechanisms, corticosteroids suppress transcription of pro-inflammatory mediators such as IL-1β, IL-*6*, and TNF*-α*, while also influencing leukocyte trafficking, antigen presentation, and lymphocyte survival in a cell-type– and context-dependent manner [52, 65].

In critical illness, corticosteroid biology extends beyond simple immunosuppression and must be interpreted within the context of neuroendocrine–immune integration [66]. Severe infection and systemic inflammation activate the Hypothalamic-Pituitary-Adrenal (HPA) axis and alter cortisol availability and signalling at the tissue level [67, 68]. The critical illness–related corticosteroid insufficiency (CIRCI) framework conceptualises inadequate cellular corticosteroid activity relative to illness severity as a consequence of dysregulated HPA signalling, altered cortisol metabolism, and tissue resistance to glucocorticoids, rather than absolute adrenal failure [54, 69]. In selected phenotypes characterised by maladaptive inflammation and escalating organ support, exogenous corticosteroids may therefore *theoretically* restore or augment anti-inflammatory signalling and improve physiological stability.

However, the same molecular pathways that underlie potential benefit also provide a mechanistic rationale for harm. Glucocorticoids can suppress antigen presentation and key effector functions in innate and adaptive immunity, with effects shaped by GR isoform expression, post-translational receptor modifications, and local regulation of steroid activity by enzymes such as 11β-hydroxysteroid dehydrogenases [52]. Moreover, critical illness itself modifies cortisol transport and metabolism, including alterations in corticosteroid-binding globulin and inflammatory regulation of cortisol clearance, complicating the prediction of tissue-level exposure and cellular responsiveness [54, 70]. Taken together, these considerations support corticosteroid use only under carefully defined conditions, as net biological effects are highly context dependent.

### Why extrapolation from immunocompetent patients is problematic

Despite a plausible mechanistic rationale, translating corticosteroid strategies from immunocompetent patients with CAP/sCAP to immunocompromised ICU populations is fundamentally constrained by structural gaps in the evidence base. Major international CAP guidelines explicitly limit their scope to adults without immunocompromising conditions, excluding patients with inherited or acquired immune deficiencies, active chemotherapy, drug-induced neutropenia, advanced HIV infection, or solid organ and hematopoietic stem cell transplantation [71]. Similarly, contemporary international sCAP guidelines list immunosuppression, including systemic corticosteroid therapy, chemotherapy, transplantation, hematologic malignancy, and advanced HIV infection, as exclusion criteria [72–77]. As a result, the populations most vulnerable to infection-related harm are systematically underrepresented in the trials that inform current practice.

Methodological features of randomised trials and meta-analyses evaluating corticosteroids in CAP further limit extrapolation. Most studies prioritise short-term endpoints, such as 28-day mortality, early clinical stability, or duration of organ support, with limited or no systematic assessment of delayed infectious complications, including opportunistic infections, viral reactivation, or *breakthrough* infections, outcomes that are particularly relevant in immunocompromised hosts [52]. Attenuation of inflammatory signals during corticosteroid therapy may further delay recognition of these complications, thereby compounding diagnostic uncertainty and failing to be captured by early trial endpoints.

Beyond trial design, expert consensus documents addressing CAP in immunocompromised adults emphasise that standard CAP algorithms are insufficient in this population due to broader pathogen spectra, higher rates of co-infection, and the need for individualised diagnostic and therapeutic strategies, factors that have historically driven exclusion from large prospective studies [78]. Heterogeneity within immunocompromised states further complicates interpretation: baseline immune defects may predominantly affect neutrophil function, antigen presentation, or T-cell–mediated immunity [79–82], each with distinct implications for how glucocorticoid-driven transcriptional programs reshape inflammation and pathogen control.

Accordingly, the central question is not whether corticosteroids are beneficial or harmful per se, but in whom, when, and under what biological and clinical conditions their use may confer net benefit. The mismatch between mechanistic plausibility, trial populations, and clinically relevant outcomes in immunocompromised ICU patients frames the uncertainty addressed in the following section, which critically examines the available clinical evidence and its limitations in this high-risk population.

## Clinical evidence

### Evidence in immunocompromised patients with pneumonia

Direct clinical evidence supporting the use of systemic corticosteroids in immunocompromised ICU patients with sCAP remains limited and fragmented. Although several contemporary randomised trials and meta-analyses have established corticosteroids as an effective adjunct in selected populations with sCAP, these data derive predominantly from immunocompetent cohorts, with immunocompromised hosts either excluded, underrepresented, or insufficiently characterised to allow reliable subgroup inference [3, 76, 77, 83]. As a result, the evidence base informing corticosteroid use in ICU-level pneumonia does not map cleanly onto the population at highest risk for infection-related harm.

The CAPE COD randomised trial demonstrated that early hydrocortisone administration in ICU patients with sCAP reduced 28-day mortality compared with placebo (25/400 [6.2%] vs. 47/395 [11.9%]) [3]. However, baseline immunosuppression was present in only a small minority of participants (approximately 6% in each group), limiting interpretability for immunocompromised ICU patients. In contrast, the REMAP-CAP adaptive platform trial evaluating a fixed 7-day course of hydrocortisone (50 mg every 6 h) in ICU patients with sCAP was stopped for futility based on a prespecified Bayesian threshold (< 5% probability of a > 20% relative reduction in mortality) [84]. Ninety-day mortality was numerically higher in the hydrocortisone group than in the control group (15% vs. 9.8%), with adjusted odds ratios crossing unity. Although this platform was not designed to specifically evaluate immunocompromised hosts, the divergent signal compared with CAPE COD underscores that corticosteroid effects in ICU pneumonia are sensitive to regimen, timing, and patient selection.

Systematic reviews and meta-analyses integrating these trials provide important context but do not close the evidence gap for immunocompromised ICU pneumonia. An updated systematic review and dose–response meta-analysis of corticosteroids in CAP incorporated recent large trials and explored regimen characteristics [12, 14]. While these syntheses support benefit signals in hospitalised CAP overall, they largely reflect immunocompetent populations and prioritise short-term outcomes, limiting their applicability to immunocompromised hosts, in whom delayed opportunistic infections and pathogen-specific risks are central to clinical decision-making.

### High-risk subgroups

Within immunocompromised pneumonia, the most robust evidence supporting adjunctive corticosteroids arises from pathogen-specific rather than syndrome-based contexts [85–87]. In AIDS-associated severe *PJP*, a double-blind, placebo-controlled trial demonstrated improved outcomes with adjunctive corticosteroids compared with placebo [88]. This landmark study provides high-grade evidence that corticosteroid benefit can be substantial when host-response biology and pathogen-specific inflammatory injury are well defined. However, this benefit does not extend uniformly across immunocompromised states. In HIV-negative immunocompromised patients with severe *P. jirovecii* pneumonia, a contemporary multicentre randomised trial of adjunctive methylprednisolone did not show a statistically significant reduction in 28-day mortality, despite numerical differences favouring corticosteroids [87, 89].

Beyond *P. jirovecii*, observational cohorts highlight substantial heterogeneity in outcomes across immunocompromised subgroups with CAP. A prospective cohort study reported marked gradients in long-term mortality associated with different immunocompromising conditions, with particularly poor outcomes among patients with advanced malignancy, chronic corticosteroid exposure, and advanced HIV infection [8]. Complementary studies in hematologic malignancy populations similarly describe distinct clinical trajectories and pathogen profiles that complicate the application of uniform adjunctive immunomodulatory strategies [90, 91].

**Fig. 2:**
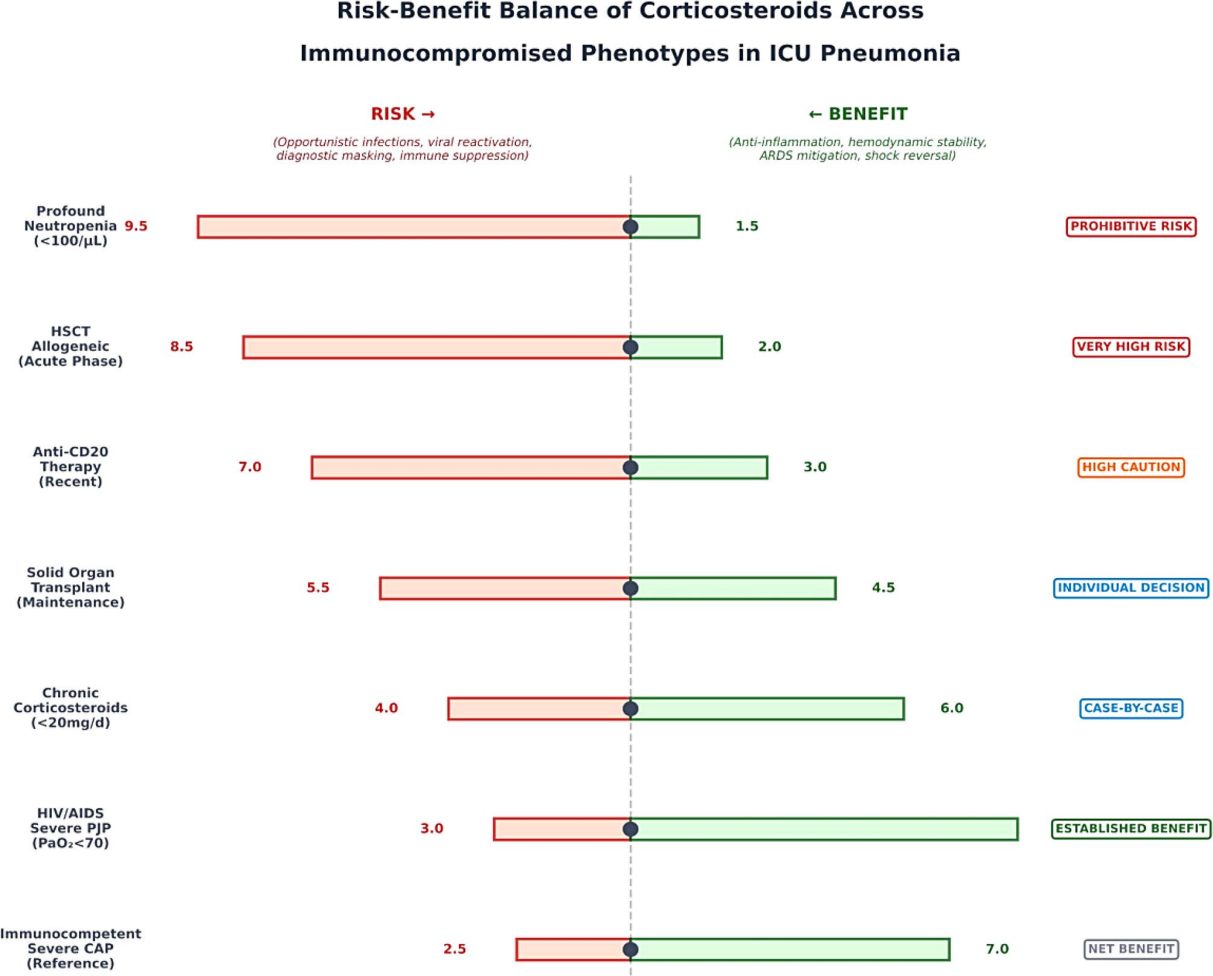
Risk-Benefit Balance of Corticosteroids Across Immunocompromised Phenotypes in ICU Pneumonia. Conceptual representation of the differential risk-benefit balance for adjunctive corticosteroid therapy across major immunocompromised subgroups admitted to the ICU with pneumonia. Risk scores (red bars, extending left from centre) represent the probability and severity of corticosteroid-associated harms, including opportunistic infections (e.g., invasive aspergillosis, Pneumocystis jirovecii pneumonia), viral reactivation (e.g., cytomegalovirus, herpes simplex virus), diagnostic masking of fever and inflammatory markers, and deepened immune suppression. Benefit scores (green bars, extending right from centre) represent the magnitude of potential benefit from anti-inflammatory effects, including attenuation of pulmonary inflammation, reduction in capillary leak, hemodynamic stabilisation, ARDS mitigation, and shock reversal. The central pivot point represents clinical equipoise, where risks and benefits are balanced. Populations with predominantly red-dominant profiles (e.g., profound neutropenia < 100/µL, allogeneic hematopoietic stem cell transplant in acute phase) face prohibitive or very high risk with minimal expected benefit, suggesting corticosteroids should generally be avoided or reserved for exceptional circumstances with intensive monitoring. Populations with intermediate or balanced profiles (e.g., solid organ transplant on maintenance immunosuppression, chronic low-dose corticosteroids) require individualised, case-by-case decision-making that incorporates specific clinical context, pathogen identification, and risk mitigation strategies. Populations with benefit-dominant profiles (e.g., HIV/AIDS with severe Pneumocystis jirovecii pneumonia [PaO₂ <70 mmHg], immunocompetent patients with severe community-acquired pneumonia) may derive net benefit when corticosteroids are used appropriately with established regimens. Scores are conceptual estimates based on synthesis of available evidence, biological plausibility, and clinical consensus, and are NOT derived from quantitative meta-analysis or head-to-head comparative trials. They serve to illustrate the profound heterogeneity of risk-benefit balance across immunocompromised phenotypes rather than provide precise numerical predictions for individual patients

Collectively, these subgroup-specific data reinforce a central message of this review: evidence supporting corticosteroids in immunocompromised pneumonia is strongest when anchored to specific host–pathogen contexts, and weakest when extrapolated across heterogeneous immunosuppressed populations. Table 2; Fig. 2 provide a comprehensive summary of the available clinical evidence for corticosteroid use across different immunocompromised subgroups and pathogen-specific contexts, highlighting the contrast between the robust evidence supporting adjunctive corticosteroids in AIDS-associated severe PJP and the substantially weaker or conflicting evidence in other immunocompromised populations.

**Table 2:** Summary of clinical evidence for corticosteroids in immunocompromised icu patients with pneumonia: pathogen-specific and population-based data

Population	Clinical Scenario	Study Design	Key Findings	Quality of Evidence	Reference
HIV/AIDS with severe PJP	Moderate-severe Pneumocystis jirovecii pneumonia (PaO₂ <70 mmHg or A-a gradient > 35 mmHg)	RCT, meta-analysis	Reduced mortality and respiratory failure with adjunctive corticosteroids	**Moderate **⊕⊕⊕○	Gagnon et al., 1990 [7]
HIV-negative immunocompromised with severe PJP	Severe Pneumocystis jirovecii pneumonia requiring ICU admission	Multicenter RCT	No significant reduction in 28-day mortality with methylprednisolone; numerical trend favouring corticosteroids not statistically significant	**Moderate **⊕⊕⊕○	Lemiale et al., 2025 [87]
Immunocompetent patients (reference)	Severe CAP requiring ICU admission	RCT (CAPE COD)	Reduced 28-day mortality (6.2% vs. 11.9%), lower intubation and vasopressor initiation rates; however, immunocompromised patients represented < 6% of cohort	**High **⊕⊕⊕⊕	Dequin et al., 2023 [3]
Immunocompetent patients (reference)	Severe CAP requiring ICU admission	Adaptive platform RCT (REMAP-CAP)	Fixed 7-day hydrocortisone stopped for futility; numerically higher 90-day mortality in hydrocortisone group (15% vs. 9.8%)	**High **⊕⊕⊕⊕	REMAP-CAP Investigators; Angus et al., 2025 [84]
General immunocompromised population	CAP requiring ICU admission in immunocompromised hosts	Retrospective cohort (MIMIC-IV)	32% of ICU CAP patients were immunocompromised; higher short- and long-term mortality; increased detection of Pneumocystis (1.1% vs. 0.3%) and Aspergillus (3.1% vs. 1.2%)	**Low **⊕⊕○○	Viñan Garcés et al., 2025 [6]
Hematologic malignancy	Pneumonia with respiratory failure	Retrospective cohorts	Inconsistent results; concern for increased invasive fungal infection rates and mortality in some cohorts	**Very low **⊕○○○	Certan et al., 2022 [90]
Solid organ transplant	CAP/HAP with shock or respiratory failure	Case series, small cohorts	Conflicting results; concern for CMV reactivation, invasive fungal infection, and graft dysfunction	**Very low **⊕○○○	Limited data
HSCT (allogeneic)	Pneumonia with respiratory failure	Retrospective cohorts	Associated with increased mortality in most studies; heightened risk of invasive aspergillosis and CMV reactivation	**Low **⊕⊕○○	Limited data

Abbreviations: CAP, community-acquired pneumonia; CMV, cytomegalovirus; HAP, hospital-acquired pneumonia; HSCT, hematopoietic stem cell transplant; ICU, intensive care unit; PJP, Pneumocystis jirovecii pneumonia; RCT, randomized controlled trial

Quality of evidence graded using modified GRADE approach: ⊕⊕⊕⊕ High (further research very unlikely to change confidence in estimate); ⊕⊕⊕○ Moderate (further research likely to have important impact); ⊕⊕○○ Low (further research very likely to have important impact); ⊕○○○ Very low (very uncertain about estimate). The strongest evidence supporting corticosteroids in immunocompromised pneumonia derives from AIDS-associated PJP; evidence is substantially weaker or conflicting across other immunocompromised subgroups, and major CAP trials systematically excluded or underrepresented immunocompromised patients

### Biological heterogeneity within immunocompromised patients

Immune profiling studies in critically ill patients demonstrate convergence toward shared patterns of immune dysfunction across diverse aetiologies, yet with substantial inter-individual variability in pathways linked to antigen presentation, lymphocyte biology, and effector function [42].

In this context, data-driven analyses of randomised CAP trials demonstrate that corticosteroid effects may cluster within biologically or clinically defined subsets rather than distribute uniformly across populations [12]. Although derived primarily from immunocompetent cohorts, this observation is particularly salient for immunocompromised ICU pneumonia, where immune-defect heterogeneity and pathogen diversity are even greater. Averaging across such variability risks obscuring meaningful benefits in selected phenotypes while underestimating harm in others.

## Practical clinical considerations in the ICU

### Circumstances in which corticosteroids should be deferred

In immunocompromised ICU patients with pneumonia, corticosteroids should be deferred when the clinical presentation raises a non-negligible suspicion for opportunistic, reactivation, or diagnostically elusive infections, particularly when microbiologic clarification has not yet been pursued. In this setting, additional immunosuppression may exacerbate diagnostic delay by blunting inflammatory signals that typically prompt timely invasive investigations, such as bronchoscopy and pathogen-directed testing, thereby increasing the risk of missed or delayed diagnoses [41].

This principle is especially relevant in patients with clinical or radiologic features compatible with invasive pulmonary aspergillosis, including ICU-associated presentations described within the AspICU framework, where systemic corticosteroid exposure is recognised as a host-level risk factor for disease development and progression [92]. Similarly, in ICU pneumonia following severe viral infections, where COVID-19-Associated Pulmonary Aspergillosis (CAPA) or Influenza-Associated Pulmonary Aspergillosis (IAPA) are plausible, corticosteroids should not be initiated in isolation from a parallel diagnostic and antifungal strategy, given the established association between corticosteroid exposure and invasive aspergillosis in these contexts [93, 94].

High-consequence scenarios further support deferral. In patients with epidemiologic exposure to *Strongyloides stercoralis*, systemic corticosteroids may precipitate hyperinfection syndrome with catastrophic outcomes, underscoring the importance of screening and risk stratification before corticosteroid initiation [95]. In addition, among hosts at risk for viral reactivation, particularly cytomegalovirus, corticosteroid exposure has been associated with adverse ICU trajectories, reinforcing the need for heightened vigilance when considering corticosteroids in this population [96].

### Principles of use

When corticosteroids are used in immunocompromised ICU patients with CAP, three conservative principles emerge from the existing evidence. First, corticosteroid exposure should be minimised. Discordant findings across ICU CAP trials suggest that dose, timing, and duration materially influence the net biological effect, supporting the use of regimens anchored to the studied protocols and early reassessment of ongoing indications rather than open-ended therapy [3, 84]. Second, corticosteroid initiation should be paired with a diagnostic strategy proportionate to opportunistic risk. In immunocompromised ICU pneumonia, opportunistic pathogens are frequently identified, and corticosteroid exposure constitutes a recognised host factor in invasive fungal disease frameworks, reinforcing the need for contemporaneous microbiologic evaluation when corticosteroids are introduced [6, 92]. Third, preventive considerations should be integrated into decision-making. Evidence supports screening for *Strongyloides* in exposed populations, attention to PJP prophylaxis in patients with cumulative corticosteroid exposure, and vigilance for viral reactivation in high-risk hosts, particularly in the ICU setting [96–98].

### Conceptual decision framework

**Fig. 3:**
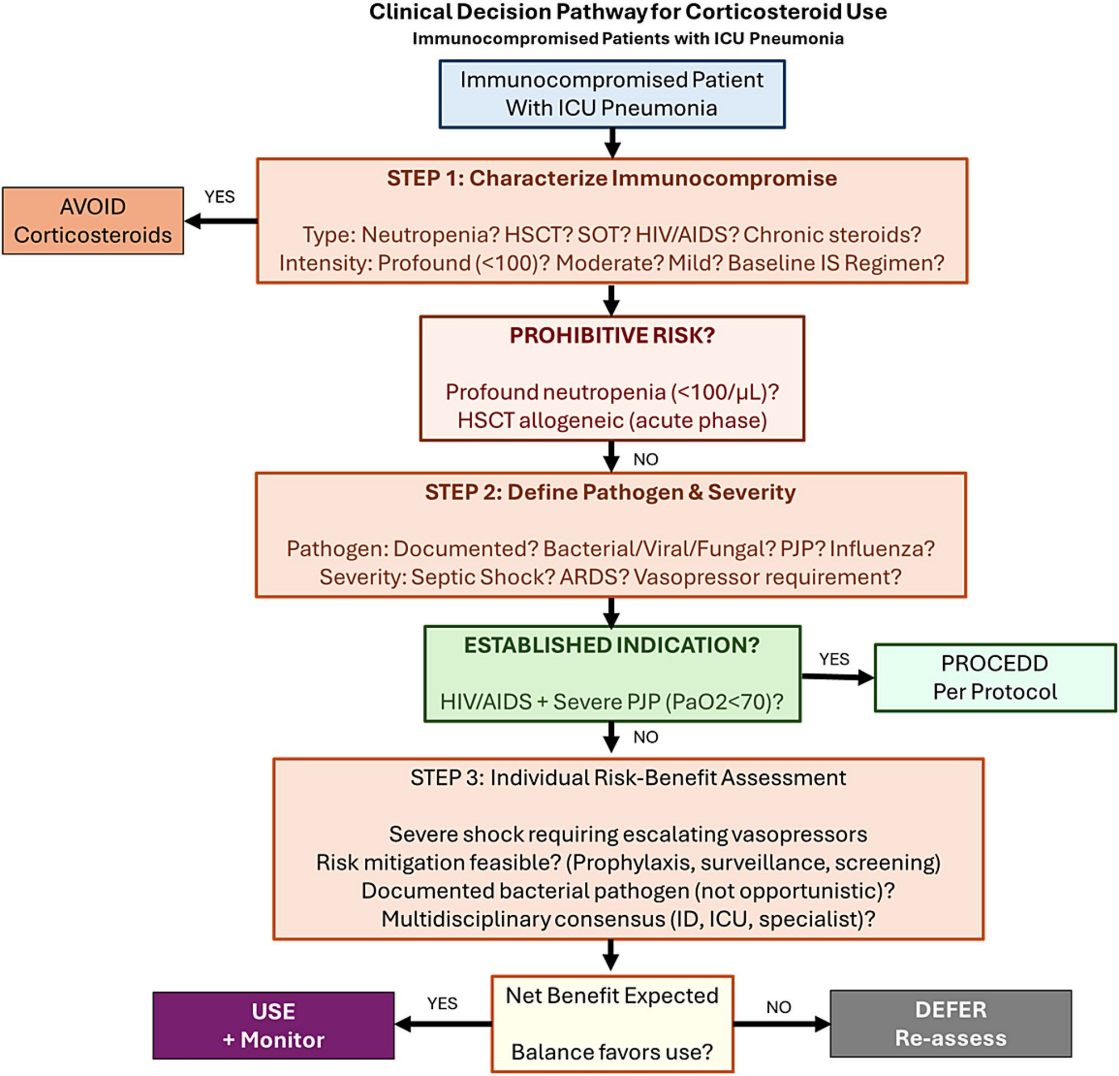
Clinical Decision Pathway for Corticosteroid Use in Immunocompromised ICU Pneumonia. Conceptual framework for structured corticosteroid decision-making in immunocompromised ICU patients with pneumonia. The pathway begins with characterisation of immunocompromise type and intensity (STEP 1), enabling identification of prohibitive risk scenarios (profound neutropenia < 100/µL, allogeneic HSCT acute phase) where corticosteroids should generally be avoided. Pathogen and severity definition (STEP 2) identifies the established benefit scenario (HIV/AIDS with severe PJP, PaO₂<70 mmHg) where corticosteroids should proceed per protocol. For all other patients, the individualised risk-benefit assessment (STEP 3) integrates shock severity, feasibility of risk mitigation, pathogen context, and multidisciplinary consensus to determine whether net benefit is expected. If benefit is expected, corticosteroids may be used with intensive monitoring; if not, therapy should be deferred with reassessment. This pathway is a conceptual framework designed to promote transparent decision-making, not a prescriptive protocol. It reflects a synthesis of limited evidence, biological plausibility, and clinical consensus rather than high-quality RCT data in immunocompromised populations

In the absence of dedicated randomised evidence, corticosteroid use in immunocompromised ICU patients with pneumonia is best approached through a transparent, structured framework that prospectively addresses key clinical and biological domains. Table 3 presents a conceptual decision-making framework organised into six interconnected domains: host characterisation; infection definition and pathogen assessment; severity assessment and therapeutic target; opportunistic risk stratification; implementation principles; and monitoring with reassessment. This framework does not constitute a prescriptive guideline but operationalises the central premise of this review, in which, when, with which regimen, and accompanied by which diagnostic or preventive bundle, while remaining consistent with the limitations of the current evidence base. Figure 3 operationalises this conceptual decision framework as a clinical pathway, illustrating the stepwise approach to corticosteroid decision-making that addresses the central questions of in whom, when, with which safeguards, and under what circumstances corticosteroids may be considered in immunocompromised ICU patients with pneumonia.

**Table 3:** Conceptual framework for corticosteroid decision-making in immunocompromised ICU patients with pneumonia

Decision Domain	Key Assessment Questions	Required Actions and Considerations	Risk Mitigation Strategies
1. Host Characterization	• What is the specific type and mechanism of immunocompromise? • What is the intensity of immune dysfunction (neutrophil count, CD4 count, immunoglobulin levels)? • What other immunosuppressive agents are being used concurrently? • What is the cumulative corticosteroid exposure (baseline dose × duration)?	• Classify type of immunocompromise (transplant, hematologic malignancy, HIV, biologic therapy, chronic steroids) • Quantify immune function with available markers • Review complete immunosuppressive medication list and calculate cumulative steroid burden	• Consult subspecialty teams (infectious diseases, haematology-oncology, transplant medicine) for immune status assessment • Characterize baseline immune deficit to estimate reserve • Consider adjusting other immunosuppressants if corticosteroids are initiated
2. Infection Definition and Pathogen Assessment	• Has microbiology been documented or pathogen-directed therapy initiated? • Have opportunistic pathogens been reasonably excluded? • Is viral coinfection present or suspected? • Is antimicrobial therapy adequate for identified or suspected pathogens?	• Obtain comprehensive microbiologic workup: blood cultures, sputum cultures, respiratory PCR panel • Measure fungal biomarkers (serum galactomannan, β-D-glucan) • Check CMV/HSV PCR in high-risk hosts (transplant, HSCT) • Ensure adequate source control and antimicrobial coverage	• Bronchoscopy with BAL if clinical deterioration or lack of expected improvement • Serial fungal surveillance in high-risk populations • Repeat imaging (CT chest) if clinical-radiological mismatch or concern for invasive fungal infection • Low threshold for invasive diagnostics given diagnostic distortion risk
3. Severity Assessment and Therapeutic Target	• Is refractory shock present despite adequate resuscitation and vasopressor support? • What is the severity of ARDS (PaO₂/FiO₂ ratio)? • Is there an established pathogen-specific indication (e.g., severe PJP in AIDS)? • Is multi-organ failure present?	• Define clear, explicit therapeutic target (shock reversal, ARDS mitigation, pathogen-specific adjunct) • Document severity scores (SOFA, APACHE II) • Consider alternative therapeutic options before adding corticosteroids	• Ensure adequate source control and antimicrobial optimization before attributing need to host response • Rule out reversible causes of shock (tamponade, tension pneumothorax, severe hypovolemia) • Document clear stopping criteria and reassessment plan
4. Opportunistic Risk Stratification	• What is the risk of CMV/HSV reactivation? • Are host factors for invasive fungal infection present? • Are there relevant geographic exposures (Strongyloides, endemic mycoses)? • Is there known microbial colonization (Aspergillus, Candida)?	• Assess viral reactivation risk based on immune status and exposure history • Identify mold exposure, structural lung disease, prior invasive fungal infection • Obtain epidemiologic history for endemic pathogens and parasites • Review prior microbiology for colonization patterns	**Screening**: • Strongyloides serology in patients from endemic areas (or empiric ivermectin) • HBsAg/anti-HBc screening in transplant or high-risk populations • TB testing if prolonged corticosteroid course anticipated **Prophylaxis**: • PJP prophylaxis if CD4 < 200, chronic high-dose steroids, or hematologic malignancy • Antifungal prophylaxis continuation in neutropenic or high-risk transplant patients • Antiviral prophylaxis/monitoring (acyclovir, ganciclovir, CMV PCR) in SOT/HSCT
5. Implementation Principles	• What is the lowest effective corticosteroid dose? • What is the shortest feasible duration? • What is the monitoring plan? • Are stopping triggers clearly defined?	• Use studied regimens when available (hydrocortisone 200 mg/day for shock; prednisone taper for PJP) • Account for baseline corticosteroid dose to avoid inadvertent dose escalation • Daily reassessment of ongoing indication • Define clear criteria for discontinuation (shock resolution, oxygenation improvement, pathogen-specific endpoint)	• Coordinate with transplant/oncology teams to adjust other immunosuppressive agents • Enhanced microbiologic surveillance: serial cultures, biomarkers, clinical monitoring • Multidisciplinary rounds with infectious diseases, critical care, and subspecialty teams • Document rationale and risk-benefit assessment in medical record
6. Monitoring and Reassessment	• Does the original indication still persist? • Have new fever or infiltrates emerged? • Is there objective clinical improvement? • Are infectious or non-infectious complications emerging?	• Daily evaluation of indication persistence and clinical trajectory • Serial biomarkers (inflammatory markers may be suppressed; trend cautiously) • Repeat cultures and imaging if clinical deterioration or failure to improve • Monitor for hyperglycaemia, secondary infections, organ dysfunction	• Early discontinuation when therapeutic target achieved (shock resolved, oxygenation improved) • Low threshold for bronchoscopy or advanced imaging if atypical course • Escalate antimicrobial therapy promptly if breakthrough infection suspected • Do not continue corticosteroids by inertia; reassess necessity daily

Abbreviations: APACHE, Acute Physiology and Chronic Health Evaluation; ARDS, acute respiratory distress syndrome; BAL, bronchoalveolar lavage; CMV, cytomegalovirus; CT, computed tomography; HBsAg, hepatitis B surface antigen; HSCT, hematopoietic stem cell transplant; HSV, herpes simplex virus; ICU, intensive care unit; PJP, Pneumocystis jirovecii pneumonia; SOFA, Sequential Organ Failure Assessment; SOT, solid organ transplant; TB, tuberculosis

This framework is not a prescriptive guideline but a conceptual tool to structure individualized clinical reasoning in the absence of high-quality direct evidence. The decision to use corticosteroids in immunocompromised ICU patients with pneumonia should be transparent, multidisciplinary, and anchored to an explicit therapeutic target. All six domains should be considered prospectively, with particular emphasis on defining opportunistic risk and implementing active mitigation strategies. Daily reassessment and early discontinuation are essential to minimize cumulative immunosuppressive burden

## Future directions

### Toward precision-oriented investigation

Research agendas in sepsis and ARDS emphasise the need for stratification by host biology, pathogen context, and treatment–response phenotypes, as well as the use of pragmatic and adaptive trial designs to identify heterogeneity of treatment effect [99]. In immunocompromised ICU pneumonia, this translates into prioritising studies that explicitly enrol immunocompromised patients, stratify by immune substrate and pathogen class, and capture outcomes particularly relevant to this population, including opportunistic infections, reactivation syndromes, and delayed complications.

### Implications for future research

The available evidence neither supports routine corticosteroid use nor categorical avoidance in immunocompromised ICU patients with sCAP. Instead, it delineates a field characterised by substantial biological heterogeneity, pathogen complexity, and structural gaps in trial design. Progress will depend on reframing the clinical question from whether corticosteroids should be used to in whom, when, under which biological conditions, and in combination with which diagnostic and preventive strategies. This reframing aligns with contemporary precision medicine frameworks in critical illness and provides a coherent agenda for generating evidence that is both clinically actionable and biologically grounded. Table 4 synthesises the major evidence gaps that constrain clinical decision-making in this population and proposes concrete research solutions aligned with precision medicine frameworks, stratified by priority level.

**Table 4:** Evidence gaps and corresponding research priorities for corticosteroid use in immunocompromised ICU pneumonia

Evidence Gap	Clinical Impact	Methodological Challenge	Proposed Research Solution	Priority Level
Systematic exclusion of immunocompromised patients from corticosteroid trials in CAP and severe CAP	Current practice relies on extrapolation from immunocompetent populations despite fundamental differences in immune biology, pathogen spectrum, and susceptibility to harm	Immunocompromised patients are perceived as high-risk and biologically heterogeneous, leading to exclusion from trials	Pragmatic randomized controlled trial enrolling immunocompromised ICU patients with pneumonia, stratified by immune substrate and pathogen context, with immunocompromised-specific outcomes (delayed infections, reactivation syndromes, 90-day mortality)	**HIGH**
Biological heterogeneity within immunocompromised populations not captured or stratified in clinical studies	Uniform one-size-fits-all corticosteroid strategies likely fail; treatment effects may cluster within biologically defined subsets, obscuring benefit in some and underestimating harm in others	No validated biomarkers or immune phenotypes for prospective stratification; immune defects vary qualitatively (innate vs. adaptive, cellular vs. humoral)	• Biomarker-guided adaptive trial design (e.g., enrolling only patients with hyperinflammatory phenotype based on IL-6, CRP thresholds) • Transcriptomic, metabolomic, and immune profiling in prospective cohorts to identify predictive phenotypes • Integration of host-response biomarkers into precision-medicine frameworks	**HIGH**
Pathogen-specific effects of corticosteroids unknown in immunocompromised ICU pneumonia	Benefit or harm likely varies by aetiology (bacterial vs. viral vs. fungal vs. polymicrobial); current trials do not stratify by pathogen class or systematically capture delayed infectious complications	Diagnostic uncertainty at enrolment; polymicrobial infections common; opportunistic pathogens may emerge during or after corticosteroid exposure	• Deep microbiological characterization in trials: BAL within 48–72 h, PCR multiplex respiratory panel, fungal biomarkers (galactomannan, β-D-glucan), CMV/HSV PCR • Pathogen-stratified analysis in observational cohorts and RCTs • Systematic capture of breakthrough infections and reactivation syndromes as trial endpoints	**MEDIUM-HIGH**
Optimal corticosteroid strategy (molecule, dose, timing, duration) undefined in immunocompromised hosts	Studies mix different corticosteroid molecules, doses, and durations, generating inconsistent and uninterpretable results; lack of standardization precludes comparison across studies	Multiple variables interact (hydrocortisone vs. dexamethasone vs. methylprednisolone; bolus vs. infusion; early vs. late initiation; short vs. prolonged courses)	Standardize protocols in future trials: define molecule, dose, administration route, timing, and duration based on studied regimens or pragmatic consensus Include dose-response and duration analyses in observational cohorts	**HIGH**
Outcomes most relevant to immunocompromised patients not systematically measured in trials	28-day mortality may not capture late harms (opportunistic infections days 30–90, viral reactivation, breakthrough fungal infections); functional outcomes and quality of life not prioritized	Trials typically powered for short-term endpoints; extended follow-up and infection-specific surveillance require additional resources	• Composite outcomes: 90-day mortality + major opportunistic infections (proven/probable IFI, CMV disease, bacteraemia) • Ventilator-free days extended to 60–90 days (capture reintubation for late infections) • Functional status at ICU and hospital discharge (FIM, EQ-5D) • Quality of life at 3–6 months	**HIGH**
Risk modifiers (baseline immunosuppression intensity, cumulative steroid burden, concurrent therapies) not granularly quantified	Cannot identify patients at prohibitive risk or those most likely to benefit; risk stratification tools lacking	Interaction between baseline immune status, concurrent immunosuppressive agents, and cumulative steroid exposure is complex and multidimensional	Prospective cohorts with detailed data on: immune function (neutrophil count, CD4, immunoglobulins), concurrent immunosuppressants, baseline and cumulative steroid dose, colonization status, and epidemiologic exposures Develop and validate risk stratification scores	**MEDIUM**
Lack of data on whether corticosteroids combined with diagnostic and prophylactic bundles alters risk-benefit balance	Uncertainty whether active screening (Strongyloides, HBV, TB), prophylaxis (PJP, antifungal, antiviral), and enhanced surveillance mitigate corticosteroid-associated risks	Difficult to standardize complex interventions; implementation variability across centres	Pragmatic trials with embedded screening and prophylaxis protocols as part of the intervention arm Compare corticosteroids plus bundle versus no corticosteroids rather than corticosteroids alone	**MEDIUM**

Abbreviations: BAL, bronchoalveolar lavage; CAP, community-acquired pneumonia; CMV, cytomegalovirus; CRP, C-reactive protein; EQ-5D, EuroQol 5-dimension questionnaire; FIM, Functional Independence Measure; HBV, hepatitis B virus; HSV, herpes simplex virus; ICU, intensive care unit; IFI, invasive fungal infection; IL-6, interleukin-6; PCR, polymerase chain reaction; PJP, Pneumocystis jirovecii pneumonia; RCT, randomized controlled trial; TB, tuberculosis

Priority levels: HIGH = foundational gap requiring urgent attention; critical to advancing the field. MEDIUM-HIGH = important gap with significant clinical impact. MEDIUM = relevant but can be addressed in parallel with higher-priority research. This table synthesizes the major structural limitations in the current evidence base and proposes concrete research solutions aligned with precision medicine frameworks in critical illness. Progress requires collaboration across international networks, integration of advanced immune profiling, and commitment to enrolling and characterizing immunocompromised populations in future trials

## Conclusions

In immunocompromised patients admitted to the ICU with sCAP, the balance between potential benefit and harm of adjunctive corticosteroid therapy differs fundamentally from that observed in immunocompetent populations. Baseline immune defects superimposed critical illness–associated immune dysregulation, and a broader spectrum of opportunistic and co-infecting pathogens collectively modify both host response and treatment risk. As a result, evidence derived from general CAP and sCAP trials cannot be directly extrapolated to this population.

Current data do not support routine corticosteroid use in immunocompromised ICU sCAP, nor do they justify categorical avoidance. Instead, corticosteroid therapy should be approached cautiously, with explicit consideration of the intended physiological target, the underlying immune substrate, cumulative immunosuppressive burden, and pathogen-specific risk. The strongest pathogen-specific evidence for adjunctive corticosteroids in immunocompromised patients pertains to PJP, where corticosteroids reduce mortality in AIDS-associated severe disease; however, this benefit has not been consistently demonstrated in HIV-negative immunocompromised hosts with PJP, reinforcing the need for individualised decision-making even within this indication. When used, corticosteroids should be administered at the lowest effective dose, for the shortest duration, and in conjunction with an appropriate diagnostic and preventive strategy. Advancing care in this high-risk population will require studies specifically designed for immunocompromised hosts, incorporating immune stratification, pathogen-informed approaches, and outcomes relevant to delayed infectious complications. Until such evidence is available, individualised, pathophysiology-informed decision-making remains essential.

## Data Availability

No datasets were generated or analysed during the current study.

## Abbreviations

ARDS: Acute respiratory distress syndrome
AspICU: Aspergillus in ICU algorithm
CAP: Community-acquired pneumonia
CAPA: COVID-19–associated pulmonary aspergillosis
CIRCI: Critical illness–related corticosteroid insufficiency
CMV: Cytomegalovirus
COVID-19: Coronavirus disease 2019
GBD: Global Burden of Disease
GLIMP: Global Initiative for Methicillin-resistant Staphylococcus aureus Pneumonia
GR: Glucocorticoid receptor
HIV: Human immunodeficiency virus
HPA: Hypothalamic–pituitary–adrenal
HSCT: Hematopoietic stem cell transplantation
IAPA: Influenza-associated pulmonary aspergillosis
ICU: Intensive care unit
IEI: Inborn errors of immunity
IFN-γ: Interferon gamma
IL: Interleukin
IPA: Invasive pulmonary aspergillosis
LOS: Length of stay
LRI: Lower respiratory infection
PJP: Pneumocystis jirovecii pneumonia
RCT: Randomized controlled trial
SARS-CoV-2: Severe acute respiratory syndrome coronavirus 2
sCAP: Severe community-acquired pneumonia
SCCM: Society of Critical Care Medicine
ESICM: European Society of Intensive Care Medicine
TNF-α: Tumour necrosis factor alpha
VFDs: Ventilator-free days

## References

[1] Reyes LF, Conway Morris A, Serrano-Mayorga C, Derde LPG, Dickson RP, Martin-Loeches I. Community-acquired pneumonia. Lancet. 2025;406(10517):2371–88. 41110447 10.1016/S0140-6736(25)01493-X

[2] Infections GBDLR, Antimicrobial Resistance C. Global burden of lower respiratory infections and aetiologies, 1990–2023: a systematic analysis for the Global Burden of Disease Study 2023. Lancet Infect Dis. 2025. 10.1016/S1473-3099(25)00689-9 41412141

[3] Dequin PF, Meziani F, Quenot JP, et al. Hydrocortisone in Severe Community-Acquired Pneumonia. N Engl J Med. 2023;388(21):1931–41. 36942789 10.1056/NEJMoa2215145

[4] Di Pasquale MF, Sotgiu G, Gramegna A, et al. Prevalence and Etiology of Community-acquired Pneumonia in Immunocompromised Patients. Clin Infect Dis. 2019;68(9):1482–93. 31222287 10.1093/cid/ciy723 PMC6481991

[5] Angus DC, Marrie TJ, Obrosky DS, et al. Severe community-acquired pneumonia: use of intensive care services and evaluation of American and British Thoracic Society Diagnostic criteria. Am J Respir Crit Care Med. 2002;166(5):717–23. 12204871 10.1164/rccm.2102084

[6] Vinan Garces AE, Sanabria-Herrera N, Duque S, et al. Severe community-acquired pneumonia in immunosuppressed patients admitted to the ICU. Respir Med. 2025;240:108014. 40020942 10.1016/j.rmed.2025.108014

[7] Cheng GS, Crothers K, Aliberti S, et al. Immunocompromised Host Pneumonia: Definitions and Diagnostic Criteria: An Official American Thoracic Society Workshop Report. Ann Am Thorac Soc. 2023;20(3):341–53. 36856712 10.1513/AnnalsATS.202212-1019ST PMC9993146

[8] Ramirez JA, Chandler TR, Furmanek SP, et al. Community-Acquired Pneumonia in the Immunocompromised Host: Epidemiology and Outcomes. Open Forum Infect Dis. 2023;10(11):ofad565. 38023559 10.1093/ofid/ofad565 PMC10676121

[9] Papon N, Nevez G, Le Gal S, et al. Fungal infections in transplant recipients: pros and cons of immunosuppressive and antimicrobial treatment. Lancet Microbe. 2021;2(1):e6–8. 35544229 10.1016/S2666-5247(20)30199-3

[10] Werbel WA, Ison MG, Angarone MP, Yang A, Stosor V. Lymphopenia is associated with late onset Pneumocystis jirovecii pneumonia in solid organ transplantation. Transpl Infect Dis. 2018;20(3):e12876. 29512868 10.1111/tid.12876 PMC11812524

[11] Martin-Loeches I, Reyes LF, Rodriguez A. Severe community-acquired pneumonia (sCAP): advances in management and future directions. Thorax. 2025;80(8):565–75. 40360263 10.1136/thorax-2024-222296

[12] Smit JM, Van Der Zee PA, Stoof SCM, et al. Predicting benefit from adjuvant therapy with corticosteroids in community-acquired pneumonia: a data-driven analysis of randomised trials. Lancet Respir Med. 2025;13(3):221–33. 39892408 10.1016/S2213-2600(24)00405-3

[13] Reyes LF, Martin-Loeches I. Corticosteroids in community-acquired pneumonia: a step forward, but questions remain. Lancet Respir Med. 2025;13(3):191–3. 39892409 10.1016/S2213-2600(24)00418-1

[14] Pitre T, Abdali D, Chaudhuri D, et al. Corticosteroids in Community-Acquired Bacterial Pneumonia: a Systematic Review, Pairwise and Dose-Response Meta-Analysis. J Gen Intern Med. 2023;38(11):2593–606. 37076606 10.1007/s11606-023-08203-6 PMC10115386

[15] Fishman JA. Infection in Organ Transplantation. Am J Transpl. 2017;17(4):856–79. 10.1111/ajt.14208 28117944

[16] Falagas ME, Manta KG, Betsi GI, Pappas G. Infection-related morbidity and mortality in patients with connective tissue diseases: a systematic review. Clin Rheumatol. 2007;26(5):663–70. 17186117 10.1007/s10067-006-0441-9

[17] Nemeth T, Sperandio M, Mocsai A. Neutrophils as emerging therapeutic targets. Nat Rev Drug Discov. 2020;19(4):253–75. 31969717 10.1038/s41573-019-0054-z

[18] Hartmann P, Herholz K, Salzberger B, Petereit HF. Unusual and severe symptomatic impairment of neutrophil function after one cycle of temozolomide in patients with malignant glioma. Ann Hematol. 2004;83(4):212–7. 14648028 10.1007/s00277-003-0802-2

[19] Agusti C, Rano A, Rovira M, et al. Inflammatory response associated with pulmonary complications in non-HIV immunocompromised patients. Thorax. 2004;59(12):1081–8. 15563709 10.1136/thx.2004.030551 PMC1746894

[20] Silvestre-Roig C, Fridlender ZG, Glogauer M, Scapini P. Neutrophil Diversity in Health and Disease. Trends Immunol. 2019;40(7):565–83. 31160207 10.1016/j.it.2019.04.012 PMC7185435

[21] Fernandez-Ruiz M, Lopez-Medrano F, Allende LM, et al. Kinetics of peripheral blood lymphocyte subpopulations predicts the occurrence of opportunistic infection after kidney transplantation. Transpl Int. 2014;27(7):674–85. 24650360 10.1111/tri.12321

[22] Liu D, Huang SY, Sun JH, et al. Sepsis-induced immunosuppression: mechanisms, diagnosis and current treatment options. Mil Med Res. 2022;9(1):56. 36209190 10.1186/s40779-022-00422-y PMC9547753

[23] George MP, Masur H, Norris KA, Palmer SM, Clancy CJ, McDyer JF. Infections in the immunosuppressed host. Ann Am Thorac Soc. 2014;11(Suppl 4):S211–20. 25148427 10.1513/AnnalsATS.201401-038PL PMC4200572

[24] Deinhardt-Emmer S, Chousterman BG, Schefold JC, et al. Sepsis in patients who are immunocompromised: diagnostic challenges and future therapies. Lancet Respir Med. 2025;13(7):623–37. 40409328 10.1016/S2213-2600(25)00124-9

[25] Chen Y, Zhang L, Wang T, Pan X, Chen D, Liu J. Characteristics of CD4 + T-cell reduction and pulmonary infections in critically ill immunocompromised patients. J Intensive Med. 2026. 10.1016/j.jointm.2025.10.007 PMC13100858 42028145

[26] Thommen DS, Koelzer VH, Herzig P, et al. A transcriptionally and functionally distinct PD-1(+) CD8(+) T cell pool with predictive potential in non-small-cell lung cancer treated with PD-1 blockade. Nat Med. 2018;24(7):994–1004. 29892065 10.1038/s41591-018-0057-z PMC6110381

[27] McLane LM, Abdel-Hakeem MS, Wherry EJ. CD8 T Cell Exhaustion During Chronic Viral Infection and Cancer. Annu Rev Immunol. 2019;37:457–95. 30676822 10.1146/annurev-immunol-041015-055318

[28] Conti-Freitas LC, Foss-Freitas MC, Mamede RC, Foss NT. Interferon-gamma and interleukin-10 production by mononuclear cells from patients with advanced head and neck cancer. Clin (Sao Paulo). 2012;67(6):587–90. 10.6061/clinics/2012(06)07 PMC3370309 22760896

[29] Hotchkiss RS, Monneret G, Payen D. Immunosuppression in sepsis: a novel understanding of the disorder and a new therapeutic approach. Lancet Infect Dis. 2013;13(3):260–8. 23427891 10.1016/S1473-3099(13)70001-X PMC3798159

[30] Xu YT, Cao ZY, Lin S, et al. Expansion of exhausted CD8 + T cells associates with increased pulmonary fungal infection risk in anti-melanoma differentiation associated gene 5 dermatomyositis. Clin Exp Rheumatol. 2024;42(2):237–45. 38153165 10.55563/clinexprheumatol/e51d3m

[31] Cossarizza A, Chang HD, Radbruch A, et al. Guidelines for the use of flow cytometry and cell sorting in immunological studies (third edition). Eur J Immunol. 2021;51(12):2708–3145. 34910301 10.1002/eji.202170126 PMC11115438

[32] Carter JT, Melcher ML, Carlson LL, Roland ME, Stock PG. Thymoglobulin-associated Cd4 + T-cell depletion and infection risk in HIV-infected renal transplant recipients. Am J Transpl. 2006;6(4):753–60. 10.1111/j.1600-6143.2006.01238.x 16539632

[33] Ng WL, Chu CM, Wu AK, Cheng VC, Yuen KY. Lymphopenia at presentation is associated with increased risk of infections in patients with systemic lupus erythematosus. QJM. 2006;99(1):37–47. 16371405 10.1093/qjmed/hci155

[34] Haessler S, Guo N, Deshpande A, et al. Etiology, Treatments, and Outcomes of Patients With Severe Community-Acquired Pneumonia in a Large U.S. Sample. Crit Care Med. 2022;50(7):1063–71. 35191410 10.1097/CCM.0000000000005498 PMC9233133

[35] Wu X, Sun T, Cai Y, et al. Clinical characteristics and outcomes of immunocompromised patients with severe community-acquired pneumonia: A single-center retrospective cohort study. Front Public Health. 2023;11:1070581. 36875372 10.3389/fpubh.2023.1070581 PMC9975557

[36] McCreery RJ, Florescu DF, Kalil AC. Sepsis in Immunocompromised Patients Without Human Immunodeficiency Virus. J Infect Dis. 2020;222(Suppl 2):S156–65. 32691837 10.1093/infdis/jiaa320

[37] Singer M, Deutschman CS, Seymour CW, et al. The Third International Consensus Definitions for Sepsis and Septic Shock (Sepsis-3). JAMA. 2016;315(8):801–10. 26903338 10.1001/jama.2016.0287 PMC4968574

[38] Seymour CW, Kennedy JN, Wang S, et al. Derivation, Validation, and Potential Treatment Implications of Novel Clinical Phenotypes for Sepsis. JAMA. 2019;321(20):2003–17. 31104070 10.1001/jama.2019.5791 PMC6537818

[39] Conway Morris A, Rynne J, Shankar-Hari M. Compartmentalisation of immune responses in critical illness: does it matter? Intensive Care Med. 2022;48(11):1617–20. 36050558 10.1007/s00134-022-06871-2 PMC9436168

[40] Khoyratty TE, Ai Z, Ballesteros I, et al. Distinct transcription factor networks control neutrophil-driven inflammation. Nat Immunol. 2021;22(9):1093–106. 34282331 10.1038/s41590-021-00968-4 PMC7611586

[41] Delano MJ, Ward PA. The immune system’s role in sepsis progression, resolution, and long-term outcome. Immunol Rev. 2016;274(1):330–53. 27782333 10.1111/imr.12499 PMC5111634

[42] Venet F, Textoris J, Blein S, et al. Immune Profiling Demonstrates a Common Immune Signature of Delayed Acquired Immunodeficiency in Patients With Various Etiologies of Severe Injury. Crit Care Med. 2022;50(4):565–75. 34534131 10.1097/CCM.0000000000005270

[43] Bergeron Y, Ouellet N, Deslauriers AM, Simard M, Olivier M, Bergeron MG. Cytokine kinetics and other host factors in response to pneumococcal pulmonary infection in mice. Infect Immun. 1998;66(3):912–22. 9488375 10.1128/iai.66.3.912-922.1998 PMC107995

[44] Duggal NA, Snelson C, Shaheen U, Pearce V, Lord JM. Innate and adaptive immune dysregulation in critically ill ICU patients. Sci Rep. 2018;8(1):10186. 29976949 10.1038/s41598-018-28409-7 PMC6033948

[45] Calfee CS, Delucchi K, Parsons PE, et al. Subphenotypes in acute respiratory distress syndrome: latent class analysis of data from two randomised controlled trials. Lancet Respir Med. 2014;2(8):611–20. 24853585 10.1016/S2213-2600(14)70097-9 PMC4154544

[46] Papathanakos G, Andrianopoulos I, Xenikakis M, et al. Clinical sepsis phenotypes in critically ill patients. Microorganisms. 2023;11(9). 10.3390/microorganisms11092165 PMC10538192 37764009

[47] Gordon AC, Alipanah-Lechner N, Bos LD, et al. From ICU Syndromes to ICU Subphenotypes: Consensus Report and Recommendations for Developing Precision Medicine in the ICU. Am J Respir Crit Care Med. 2024;210(2):155–66. 38687499 10.1164/rccm.202311-2086SO PMC11273306

[48] Investigators R-C, Hills TE, Lorenzi E, et al. Simvastatin in Critically Ill Patients with Covid-19. N Engl J Med. 2023;389(25):2341–54. 37888913 10.1056/NEJMoa2309995 PMC10755839

[49] Moreno G, Carbonell R, Martin-Loeches I, et al. Corticosteroid treatment and mortality in mechanically ventilated COVID-19-associated acute respiratory distress syndrome (ARDS) patients: a multicentre cohort study. Ann Intensive Care. 2021;11(1):159. 34825976 10.1186/s13613-021-00951-0 PMC8617372

[50] Stahn C, Buttgereit F. Genomic and nongenomic effects of glucocorticoids. Nat Clin Pract Rheumatol. 2008;4(10):525–33. 18762788 10.1038/ncprheum0898

[51] Barnes PJ. Corticosteroid effects on cell signalling. Eur Respir J. 2006;27(2):413–26. 16452600 10.1183/09031936.06.00125404

[52] Cain DW, Cidlowski JA. Immune regulation by glucocorticoids. Nat Rev Immunol. 2017;17(4):233–47. 28192415 10.1038/nri.2017.1 PMC9761406

[53] Kono M, Yamaki H, Komatsuda H, et al. IL-2 complex recovers steroid-induced inhibition in immunochemotherapy for head and neck cancer. Transl Oncol. 2022;18:101358. 35123188 10.1016/j.tranon.2022.101358 PMC8819385

[54] Annane D, Pastores SM, Arlt W, et al. Critical illness-related corticosteroid insufficiency (CIRCI): a narrative review from a Multispecialty Task Force of the Society of Critical Care Medicine (SCCM) and the European Society of Intensive Care Medicine (ESICM). Intensive Care Med. 2017;43(12):1781–92. 28940017 10.1007/s00134-017-4914-x

[55] Dickson RP, Erb-Downward JR, Martinez FJ, Huffnagle GB. The Microbiome and the Respiratory Tract. Annu Rev Physiol. 2016;78:481–504. 26527186 10.1146/annurev-physiol-021115-105238 PMC4751994

[56] Dickson RP, Huffnagle GB. The Lung Microbiome: New Principles for Respiratory Bacteriology in Health and Disease. PLoS Pathog. 2015;11(7):e1004923. 26158874 10.1371/journal.ppat.1004923 PMC4497592

[57] Bustos IG, Martin-Loeches I, Acosta-Gonzalez A, Chotirmall SH, Dickson RP, Reyes LF. Exploring the complex relationship between the lung microbiome and ventilator-associated pneumonia. Expert Rev Respir Med. 2023;17(10):889–901. 37872770 10.1080/17476348.2023.2273424

[58] Hartmann JE, Albrich WC, Dmitrijeva M, Kahlert CR. The Effects of Corticosteroids on the Respiratory Microbiome: A Systematic Review. Front Med (Lausanne). 2021;8:588584. 33777968 10.3389/fmed.2021.588584 PMC7988087

[59] Toraldo DM, Conte L. Influence of the Lung Microbiota Dysbiosis in Chronic Obstructive Pulmonary Disease Exacerbations: The Controversial Use of Corticosteroid and Antibiotic Treatments and the Role of Eosinophils as a Disease Marker. J Clin Med Res. 2019;11(10):667–75. 31636780 10.14740/jocmr3875 PMC6785281

[60] Flanagan JL, Brodie EL, Weng L, et al. Loss of bacterial diversity during antibiotic treatment of intubated patients colonized with Pseudomonas aeruginosa. J Clin Microbiol. 2007;45(6):1954–62. 17409203 10.1128/JCM.02187-06 PMC1933106

[61] Iwai S, Huang D, Fong S, et al. The lung microbiome of Ugandan HIV-infected pneumonia patients is compositionally and functionally distinct from that of San Franciscan patients. PLoS ONE. 2014;9(4):e95726. 24752365 10.1371/journal.pone.0095726 PMC3994144

[62] Vidaur L, Guridi A, Leizaola O, et al. Respiratory dysbiosis as prognostic biomarker of disease severity for adults with community-acquired pneumonia requiring mechanical ventilation. Pneumonia (Nathan). 2025;17(1):10. 40320531 10.1186/s41479-025-00163-1 PMC12051328

[63] Cheng YN, Chen GT, Huang WC, et al. Lung microbiome signatures and explainable predictive modeling of glucocorticoid response in severe community acquired pneumonia. Front Microbiol. 2025;16:1706432. 41395471 10.3389/fmicb.2025.1706432 PMC12698617

[64] Stahn C, Lowenberg M, Hommes DW, Buttgereit F. Molecular mechanisms of glucocorticoid action and selective glucocorticoid receptor agonists. Mol Cell Endocrinol. 2007;275(1–2):71–8. 17630118 10.1016/j.mce.2007.05.019

[65] Gerber AN, Newton R, Sasse SK. Repression of transcription by the glucocorticoid receptor: A parsimonious model for the genomics era. J Biol Chem. 2021;296:100687. 33891947 10.1016/j.jbc.2021.100687 PMC8141881

[66] Cavagnini F, Croci M, Putignano P, Petroni ML, Invitti C. Glucocorticoids and neuroendocrine function. Int J Obes Relat Metab Disord. 2000;24(Suppl 2):S77–9. 10997615 10.1038/sj.ijo.0801284

[67] Langouche L, Van den Berghe G. Hypothalamic-pituitary hormones during critical illness: a dynamic neuroendocrine response. Handb Clin Neurol. 2014;124:115–26. 25248583 10.1016/B978-0-444-59602-4.00008-3

[68] Van den Berghe G, Teblick A, Langouche L, Gunst J. The hypothalamus-pituitary-adrenal axis in sepsis- and hyperinflammation-induced critical illness: Gaps in current knowledge and future translational research directions. EBioMedicine. 2022;84:104284. 36162206 10.1016/j.ebiom.2022.104284 PMC9519475

[69] Mazzeo AT, Guaraldi F, Filippini C, et al. Activation of pituitary axis according to underlying critical illness and its effect on outcome. J Crit Care. 2019;54:22–9. 31326617 10.1016/j.jcrc.2019.07.006

[70] Beishuizen A, Thijs LG, Vermes I. Patterns of corticosteroid-binding globulin and the free cortisol index during septic shock and multitrauma. Intensive Care Med. 2001;27(10):1584–91. 11685298 10.1007/s001340101073

[71] Metlay JP, Waterer GW, Long AC, et al. Diagnosis and Treatment of Adults with Community-acquired Pneumonia. An Official Clinical Practice Guideline of the American Thoracic Society and Infectious Diseases Society of America. Am J Respir Crit Care Med. 2019;200(7):e45–67. 31573350 10.1164/rccm.201908-1581ST PMC6812437

[72] Martin-Loeches I, Torres A, Nagavci B, et al. ERS/ESICM/ESCMID/ALAT guidelines for the management of severe community-acquired pneumonia. Eur Respir J. 2023;61(4). 10.1183/13993003.00735-2022 37012080

[73] Snijders D, Daniels JM, de Graaff CS, van der Werf TS, Boersma WG. Efficacy of corticosteroids in community-acquired pneumonia: a randomized double-blinded clinical trial. Am J Respir Crit Care Med. 2010;181(9):975–82. 20133929 10.1164/rccm.200905-0808OC

[74] Meijvis SC, Hardeman H, Remmelts HH, et al. Dexamethasone and length of hospital stay in patients with community-acquired pneumonia: a randomised, double-blind, placebo-controlled trial. Lancet. 2011;377(9782):2023–30. 21636122 10.1016/S0140-6736(11)60607-7

[75] Blum CA, Nigro N, Briel M, et al. Adjunct prednisone therapy for patients with community-acquired pneumonia: a multicentre, double-blind, randomised, placebo-controlled trial. Lancet. 2015;385(9977):1511–8. 25608756 10.1016/S0140-6736(14)62447-8

[76] Torres A, Sibila O, Ferrer M, et al. Effect of corticosteroids on treatment failure among hospitalized patients with severe community-acquired pneumonia and high inflammatory response: a randomized clinical trial. JAMA. 2015;313(7):677–86. 25688779 10.1001/jama.2015.88

[77] Meduri GU, Shih MC, Bridges L, et al. Low-dose methylprednisolone treatment in critically ill patients with severe community-acquired pneumonia. Intensive Care Med. 2022;48(8):1009–23. 35723686 10.1007/s00134-022-06684-3 PMC9208259

[78] Ramirez JA, Musher DM, Evans SE, et al. Treatment of Community-Acquired Pneumonia in Immunocompromised Adults: A Consensus Statement Regarding Initial Strategies. Chest. 2020;158(5):1896–911. 32561442 10.1016/j.chest.2020.05.598 PMC7297164

[79] Meng F, Zhu C, Zhu C, et al. Epidemiology and pathogen characteristics of infections following solid organ transplantation. J Appl Microbiol. 2024;135(12). 10.1093/jambio/lxae292 39567858

[80] Gao Q, Wang T, Tan S, Tian Y, Zhang A. Outcomes and risk factors in HIV-positive patients with sepsis: a retrospective study. Eur J Med Res. 2025;30(1):494. 40537837 10.1186/s40001-025-02753-7 PMC12178050

[81] Etienne C, Vilcu AM, Finet F, et al. Incidence of serious respiratory tract infections and associated characteristics in a population exposed to immunosuppressive therapies: a register-based population study. BMC Infect Dis. 2024;24(1):1184. 39434000 10.1186/s12879-024-10039-2 PMC11492539

[82] He J, Li Z. Dilemma of immunosuppression and infection risk in systemic lupus erythematosus. Rheumatology (Oxford). 2023;62(Suppl 1):i22–9. 36987605 10.1093/rheumatology/keac678 PMC10050939

[83] Confalonieri M, Urbino R, Potena A, et al. Hydrocortisone infusion for severe community-acquired pneumonia: a preliminary randomized study. Am J Respir Crit Care Med. 2005;171(3):242–8. 15557131 10.1164/rccm.200406-808OC

[84] Investigators R-C, Angus DC. Effect of hydrocortisone on mortality in patients with severe community-acquired pneumonia: The REMAP-CAP Corticosteroid Domain Randomized Clinical Trial. Intensive Care Med. 2025;51(4):665–80. 40261382 10.1007/s00134-025-07861-w PMC12055926

[85] Moreno G, Rodriguez A, Reyes LF, et al. Corticosteroid treatment in critically ill patients with severe influenza pneumonia: a propensity score matching study. Intensive Care Med. 2018;44(9):1470–82. 30074052 10.1007/s00134-018-5332-4 PMC7095489

[86] Dupuis C, Timsit JF, Domitile J, et al. Corticosteroids in immunocompromised ICU patients with severe COVID-19: a multicenter retrospective study. Sci Rep. 2025;15(1):27252. 40715278 10.1038/s41598-025-10864-8 PMC12297506

[87] Lemiale V, Resche-Rigon M, Zerbib Y, et al. Adjunctive corticosteroids in non-AIDS patients with severe Pneumocystis jirovecii pneumonia (PIC): a multicentre, double-blind, randomised controlled trial. Lancet Respir Med. 2025;13(9):800–8. 40652952 10.1016/S2213-2600(25)00125-0

[88] Gagnon S, Boota AM, Fischl MA, Baier H, Kirksey OW, La Voie L. Corticosteroids as adjunctive therapy for severe Pneumocystis carinii pneumonia in the acquired immunodeficiency syndrome. A double-blind, placebo-controlled trial. N Engl J Med. 1990;323(21):1444–50. 2233916 10.1056/NEJM199011223232103

[89] Gramegna A, Sotgiu G, Di Pasquale M, et al. Atypical pathogens in hospitalized patients with community-acquired pneumonia: a worldwide perspective. BMC Infect Dis. 2018;18(1):677. 30563504 10.1186/s12879-018-3565-z PMC6299604

[90] Certan M, Garcia Garrido HM, Wong G, Heijmans J, Grobusch MP, Goorhuis A. Incidence and Predictors of Community-Acquired Pneumonia in Patients With Hematological Cancers Between 2016 and 2019. Clin Infect Dis. 2022;75(6):1046–53. 35195716 10.1093/cid/ciac005 PMC9522390

[91] Azoulay E, Zafrani L, Nates J, et al. Advances in the critical care management for patients with hematological malignancies. Blood Rev. 2025;74:101306. 40603200 10.1016/j.blre.2025.101306

[92] Blot SI, Taccone FS, Van den Abeele AM, et al. A clinical algorithm to diagnose invasive pulmonary aspergillosis in critically ill patients. Am J Respir Crit Care Med. 2012;186(1):56–64. 22517788 10.1164/rccm.201111-1978OC

[93] Verweij PE, Rijnders BJA, Bruggemann RJM, et al. Review of influenza-associated pulmonary aspergillosis in ICU patients and proposal for a case definition: an expert opinion. Intensive Care Med. 2020;46(8):1524–35. 32572532 10.1007/s00134-020-06091-6 PMC7306567

[94] Koehler P, Cornely OA, Bottiger BW, et al. COVID-19 associated pulmonary aspergillosis. Mycoses. 2020;63(6):528–34. 32339350 10.1111/myc.13096 PMC7267243

[95] Bisoffi Z, Buonfrate D, Montresor A, et al. Strongyloides stercoralis: a plea for action. PLoS Negl Trop Dis. 2013;7(5):e2214. 23675546 10.1371/journal.pntd.0002214 PMC3649953

[96] Papazian L, Hraiech S, Lehingue S, et al. Cytomegalovirus reactivation in ICU patients. Intensive Care Med. 2016;42(1):28–37. 26424680 10.1007/s00134-015-4066-9 PMC7095171

[97] Hartman ES, Cavallazzi R. Pulmonary infections in patients receiving corticosteroids and other immunomodulators. Semin Respir Crit Care Med. 2025. 10.1055/a-2767-2557 41475423

[98] Buonfrate D, Requena-Mendez A, Angheben A, et al. Accuracy of molecular biology techniques for the diagnosis of Strongyloides stercoralis infection-A systematic review and meta-analysis. PLoS Negl Trop Dis. 2018;12(2):e0006229. 29425193 10.1371/journal.pntd.0006229 PMC5823464

[99] Shah FA, Meyer NJ, Angus DC, et al. A Research Agenda for Precision Medicine in Sepsis and Acute Respiratory Distress Syndrome: An Official American Thoracic Society Research Statement. Am J Respir Crit Care Med. 2021;204(8):891–901. 34652268 10.1164/rccm.202108-1908ST PMC8534611

